# Effect of temperature acclimation period on upper thermal tolerance in a terrestrial salamander

**DOI:** 10.7717/peerj.20775

**Published:** 2026-02-03

**Authors:** Sandra C. Valderrama Robles, Molly G. Russell, Carl D. Anthony, James I. Watling

**Affiliations:** 1 Department of Biology, John Carroll University, University Heights, OH, United States of America; 2 Research Department, Holden Forests & Gardens, Kirtland, OH, United States of America; 3 Department of Biology, Wichita State University, Wichita, KS, United States of America

**Keywords:** Climate change, Thermal acclimation, Eastern Red-backed Salamander, Critical thermal maximum (CT_max_), Phenotypic plasticity

## Abstract

**Background:**

Physiological traits, such as the critical thermal maximum (CT_max_, defined as an individual’s upper thermal tolerance limit), can be important for understanding species’ vulnerability to climate and habitat change. A separate trait, thermal acclimation capacity, is defined as the physiological adjustment of organisms to temperature variation, which can influence phenotypic traits such as CT_max_. The relationship between acclimation capacity and CT_max_ has been widely studied in ectotherms like fish, amphibians, and reptiles, and it is generally observed that CT_max_ increases with higher acclimation temperatures. However, there is a lack of information about whether amphibians respond differently to long- versus short-term acclimation. Understanding thermal acclimation capacity under rapid environmental change is important, as high acclimation capacity may reduce vulnerability. Here, we evaluated the thermal acclimation capacity of the Eastern Red-backed Salamander, *Plethodon cinereus*, in response to short- and long-term acclimation treatments in a laboratory setting.

**Methods:**

We exposed salamanders to three different treatments: control animals were maintained at 15 °C for 30 days; animals in the short-term acclimation group were maintained at 15 °C for 28 days, and 23 °C for 48 hours before testing CT_max_; and animals in the long-term acclimation group were maintained at 23 °C for 30 days. We measured the CT_max_ of all animals at the end of the experiment to determine whether tolerance to high temperatures varied depending on the length of exposure to warm conditions.

**Results:**

Although we observed a slight increase in CT_max_ from the control treatment to the short-term (+0.93 °C) and long-term (+0.98 °C) acclimation treatments, the difference in CT_max_ between acclimation treatments was small (0.05 °C), and none of the differences were statistically significant.

**Discussion:**

Several factors may explain the low variation in CT_max_ described in our study, including phylogenetic conservation of upper thermal limits, or a lack of sufficient temperature differences in our treatments to elicit a physiological response. Regardless, our results provide limited evidence that different acclimation periods affect the degree of phenotypic plasticity in CT_max_ in *Plethodon cinereus*.

## Introduction

Ectothermic species rely on external factors to regulate their body temperature (Deutsch et al., 2008) and, therefore, performance, through thermoregulatory behavior, geographical range shift, or microhabitat selection (Navas, 1996; Brattstrom, 1979; Nowakowski et al., 2017; Gaudenti et al., 2021). Species in temperate zones experience highly variable thermal conditions, including long-term variation mediated by seasonal changes, and short-term variation resulting from diel changes (Sunday, Bates & Dulvy, 2011; Young & Gifford, 2013; Kefford et al., 2022). Many temperate regions experience fluctuations between 20 °C and −20 °C across different seasons, with less variable but still notable temperature fluctuation throughout the day (Sunday, Bates & Dulvy, 2011). Such thermal variation is associated with consistent geographical differences in species’ physiological traits. For example, a meta-analysis evaluating thermal tolerance breadths (the difference between the extreme high and low temperatures a species can tolerate) of 341 ectotherm species worldwide found that thermal tolerance breadths were greater for temperate species than for tropical species that experience more uniform ambient temperatures (Sunday, Bates & Dulvy, 2011). Thus, organisms exposed to variable temperatures may have a physiological advantage in novel environments, reducing their sensitivity to thermal conditions (Young & Gifford, 2013; Riddell et al., 2018).

Thermal acclimation capacity is an organism’s phenotypically plastic physiological adjustment to temperature variation (Markle & Kozak, 2018). Organisms with high acclimation capacity have the ability to adjust relatively rapidly to novel thermal conditions, which may confer an advantage when responding to the warm and thermally variable conditions caused by climate change and habitat modification (Riddell et al., 2018). Acclimation responses can influence various traits, including the critical thermal maximum (CT_max_) (Stillman, 2003; Young & Gifford, 2013), standard metabolic rate (SMR) (Markle & Kozak, 2018; Riddell et al., 2018), and performance (Marvin, 2003; MacLean et al., 2017a). CT_max_ is a thermal trait representing the highest temperature an individual can tolerate, beyond which locomotor function fails (Cowles & Bogert, 1944). Although substantial literature suggests that CT_max_ is less influenced by local environmental conditions than CT_min_ (the lowest temperature an individual can tolerate) (Brett, 1956; Sunday, Bates & Dulvy, 2011; Muñoz et al., 2014), other studies suggest that CT_max_ is a somewhat plastic trait that can vary at least in part with local thermal conditions, suggesting the possibility of short-term acclimation to a changing thermal environment (Lowe & Vance, 1955; Hutchison, 1961; Pintanel et al., 2019). For instance, the CT_max_ of the temperate salamander *Desmognathus brimleyorum* was 0.7 ° C higher when exposed to variable temperatures compared with a more homogeneous temperature treatment (Young & Gifford, 2013). In another study, thermal tolerances increased with life stages in European common frogs when exposed to different acclimation temperatures (*i.e.,* 14, 18, 25, and 28 °C) (Ruthsatz et al., 2022).

Indeed, the relationship between acclimation capacity and CT_max_ has been widely studied in ectotherms like insects (MacLean et al., 2017a; MacLean et al., 2017b), fish (Doudoroff, 1942), frogs (Davenport & Castle, 1895), and salamanders (McFarland, 1955; Markle & Kozak, 2018; Messerman, Turrell & Leal, 2022; Zhao et al., 2022). This body of research is variable in its findings but indicates that ectothermic organisms can sometimes express significant phenotypic plasticity in CT_max_ after exposure to different temperatures. For example, a study that exposed two temperate salamander species (*Ambystoma maculatum* and *A. opacum*) to different acclimation treatments (19, 27, and 30.5 °C) found that CT_max_ increased approximately 1.5 °C in the treatment at 27 °C compared to 19 °C, but showed no difference between the 27 °C and 30.5 °C treatments (Messerman, Turrell & Leal, 2022).

The study of the effects of acclimation period (*e.g.*, long- and short-term acclimation) has primarily been focused on insects (Karl et al., 2014; MacLean et al., 2017a; Khurshid et al., 2022). For instance, *Drosophila melanogaster* exposed to a constant temperature of 15, 19, or 31 °C for long-term acclimation treatments (through development) experienced an increase in CT_max_ at a rate of 0.05 °C/1 °C increase in acclimation temperature, whereas there was no effect in the short-term treatments (MacLean et al., 2017a). However, we need additional data from more species, because environmental changes such as habitat loss and modification can rapidly alter the structural complexity of vegetation, making it harder for ectotherms to find cool sites that provide refuge from high temperatures (Tuff, Tuff & Davies, 2016; Nowakowski et al., 2018; De Frenne et al., 2021). Furthermore, habitat degradation is occurring against the backdrop of climate change, exacerbating the vulnerabilities that ectotherms face when confronted with warmer and more variable thermal landscapes (González-del-Pliego et al., 2020; Cordier et al., 2021). This makes it even more urgent to understand the strategies used by species to persist in the face of rapid environmental change. Given that amphibians are the most threatened group of terrestrial vertebrates and susceptible to the thermal variability associated with landscape and climate change (Luedtke et al., 2023), understanding long- and short-term acclimation capacity in amphibians is key to assessing their vulnerability to changing thermal environments and whether they can adapt to rapid temperature changes in nature.

In this study, we evaluated the effects of short- and long-term acclimation to warm temperatures on critical thermal maxima for the Eastern Red-backed Salamander, *Plethodon cinereus*. This terrestrial, lungless amphibian is found throughout temperate forested habitats in eastern North America (Petranka, 1998) and is exposed to a wide range of temperatures across variable spatial and temporal scales (Carroll & Carroll, 2025). The aim of our study was to assess plasticity in the CT_max_ of *Plethodon cinereus* exposed to warm temperatures for different periods of time, to understand whether extended exposure to warm conditions results in higher thermal tolerance than short-term exposure to the same warm temperatures.

## Materials & Methods

### Collection and maintenance of the salamanders

In Ohio, USA, where this study took place, many populations of *P. cinereus* are polymorphic for dorsal coloration (striped *versus* unstriped; Pfingsten & Walker, 1978). The different color morphs of *P. cinereus* differ in numerous ecological, behavioral, and physiological dimensions (reviewed in Fisher-Reid et al., 2024), the most salient of which is that the unstriped morph is more associated with warmer and drier conditions than the striped morph (Lotter & Scott Jr, 1977; Anthony, Venesky & Hickerson, 2008). For this reason, we only collected striped individuals in this study. In general, and at our study site, *P. cinereus* are most active in the spring and autumn, when temperatures averaged about 13.6 °C, with fewer observations in the summer months when mean temperatures were around 19.5 °C (Taub, 1961; Anthony, Venesky & Hickerson, 2008; Anthony & Pfingsten, 2013). Temperatures approaching or exceeding 20 °C may be thermally stressful (Homyack, Hass & Hopkins et al., 2010), particularly for striped *P. cinereus* (Moreno, 1989).

Salamanders were collected in a forested area in northern Summit County, Ohio (41.23, −81.54), in October 2022 with permit approval (Ohio Division of Wildlife Permit number SC220170). We collected 65 adult striped *P. cinereus* (mean SVL∼42.9 mm) by overturning rocks and logs during daylight hours. We made no preference between males and females. Each salamander was transported in plastic vials with moist paper towels. Salamanders were housed in a square (18 × 18 × 5.4 cm) plastic container with two soaked paper towels, which were changed weekly. Salamander containers were cleaned once a week, and individuals were fed twice a week with wingless fruit flies (*Drosophila melanogaster*). All animals were housed in incubators with suitable temperature, humidity conditions and a common photoperiod (14L:10D).

All experimental procedures and laboratory housing were approved by John Carroll University’s Institutional Animal Care and Use Committee (IACUC number 2301). Upon completion of experimental trials, all salamanders were euthanized with tricaine methanesulfonate (MS-222) followed standard humane procedures. The sampling and manipulation of salamanders followed the ARROW guidelines (Field et al., 2020).

### Acclimation treatments

All salamanders were held in incubators at 15 °C for five days before starting the experiments, roughly equivalent to the temperatures in the field at the time of capture. We measured snout-to-vent length (SVL) and mass to estimate body condition. Salamanders were assigned to one of the three treatments, with the constraint that the treatments had roughly equal numbers of male and female individuals.

In the long-term acclimation treatment (*n* = 21), salamanders were housed in a single incubator maintained at a warm temperature (23 °C) for 30 days. For the short-term acclimation treatment (*n* = 22), salamanders were housed in an incubator set to 15 °C for 28 days, and then the temperature was increased to 23 °C for 48 h. The third treatment was the control (*n* = 22), in which salamanders were housed in an incubator maintained at 15 °C for 30 days. To minimize the potential for a confounding incubator effect, halfway through the experiment (day 15), we quickly moved all salamanders in each of the three treatments to different incubators, resetting the incubator temperature to maintain treatment conditions. The transfer of animals and temperature reset took less than ten minutes, with little potential for this change to meaningfully impact the acclimation treatments.

### Thermal analyses

After a month of acclimation treatments, we measured the critical thermal maxima for each individual. Before measuring CT_max_, all individuals were relabeled by a researcher not involved in the thermal trials (JIW) so that trials were conducted without knowledge of the treatment from which individuals were drawn.

Salamanders were warmed in individual glass Petri dishes containing one mm of water and floating in a water bath (11L). The initial temperature in the water bath ranged from 20.0–22.5 °C and was raised by approximately 0.3 °C per minute (Lutterschmidt et al., 1998). Each individual was monitored continuously to prevent overheating or death. At the first signs of the onset of spasms, individuals were turned on their backs to test for the righting response (*e.g.*, (Navas, 1997). The test ended when the individuals lost their righting response, at which point the individual’s body temperature (CT_max_) was recorded. The CT_max_ was recorded with a thermocouple on the ventral side of the individuals (Navas, Gomes & Carvalhoc, 2008; Navas et al., 2013). The recovery of the animals after the CT_max_ test was carried out by placing the individuals in a container with a soaked towel at room temperature (Catenazzi, Lehr & Vredenburgi, 2014).

### Statistical analysis

Previous research with amphibians and reptiles has shown that warm temperatures can be associated with decreased body condition (Moldowan, Glenn & Njal, 2022), and that CT_max_ often increases with an individual’s body size (Claunch et al., 2020). Because body condition may affect CT_max_ and/or respond to the thermal treatments we used in our study, we extracted the residuals from a regression between individual SVL and mass and included this as a covariate in our analysis.

Because there is a natural order to the levels of our acclimation treatment variable, we used an ordered analysis of variance (ANOVA) with CT_max_ as the response variable and three predictor variables: acclimation treatment, sex, and body condition. We first ran an ANOVA including all possible interactions among predictor variables. If no significant interactions were detected, we re-ran the ANOVA with additive terms for each of the predictor variables. If sex and/or body condition were not significant, they were removed from the model, and the ANOVA was re-run with only the acclimation treatment variable. In addition to the tests of main effects of the predictors, we also tested for a linear (ordered) trend in the response of CT_max_ to the acclimation treatments. Because our hypothesis of interest focuses on the difference in CT_max_ between short- and long-term acclimation, we used Tukey’s honestly significant difference post-hoc test to compare mean CT_max_ among the three levels of the acclimation treatment. Data were checked for normality using the Shapiro–Wilks test, the assumption of equal variance was evaluated using Levene’s test, and we tested for CT_max_ outliers using the interquartile range criterion. All variables met normality and equal variance assumptions, but the two lowest CT_max_ values were flagged as outliers and removed from the analysis. Statistical significance was assessed at *p* < 0.05, and all analyses were performed in the R statistical environment version 4.1.2 (R Core Team, 2021).

## Results

### Critical thermal maxima (CT_max_)

**Figure 1:**
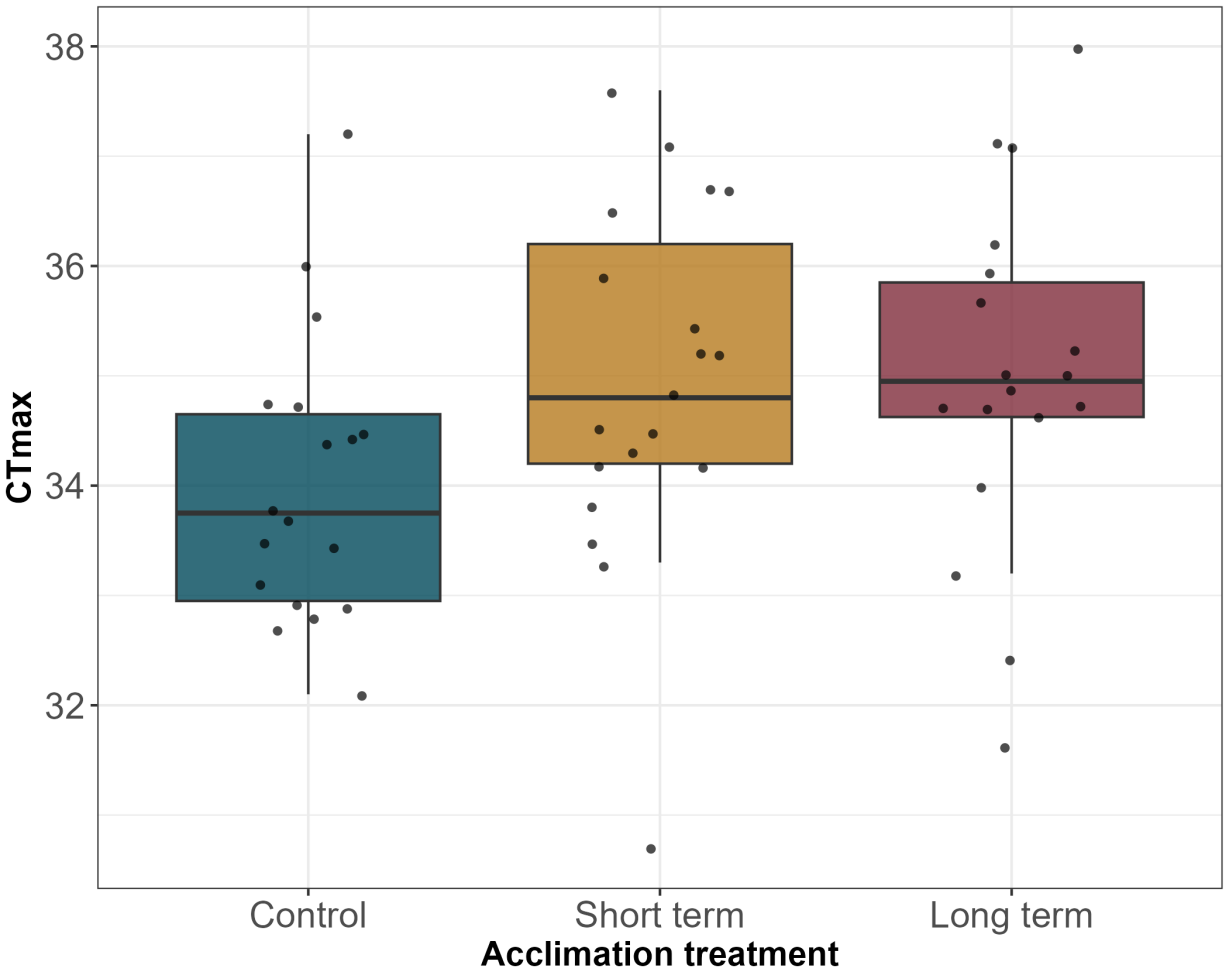
Relationship between CT_max_ and treatment acclimations. Each box represents a treatment. The blue box represents the control treatment (15 ° C (30 days)), the yellow box represents short-term treatment (15 ° C (28 days)-23 ° C (48 h)), and the red box represents long-term treatment (23 ° C (30 days)).

The mean CT_max_ was 34.02 °C (±1.30 SD, 95% CI [33.4–34.7 °C]) for the control group, 34.95 °C (±1.63 SD, 95% CI [34.2–35.7 °C]) for short-term acclimation, and 35.00 °C (±1.60 SD, 95% CI [34.2–35.8 °C]) for long-term acclimation. None of the interaction terms were significant in the model including interactions (all *p* > 0.57), and neither body condition nor sex were significant in the additive model (*P* = 0.30 for body condition and *p* = 0.87 for sex). We observed a slightly non-significant effect of acclimation period on CT_max_ (*F*_2,52_ = 2.42, *p* = 0.099), which included a slightly non-significant linear trend in mean CT_max_ across the ordered treatment levels (*F*_1,52_ = 3.77, *p* = 0.06; Fig. 1). Thus, there was a tendency for CT_max_ to increase somewhat as salamanders experienced longer exposure to a high temperature, although this trend was non-significant. However, the post-hoc test indicated that all pairwise comparisons of CT_max_ across levels of the acclimation treatment were non-significant (control-short-term *p* = 0.16; control-long-term *p* = 0.14; short-term-long-term *p* = 0.99). We note that the key comparison underlying our hypothesis is the one comparing CT_max_ between short- and long-term acclimation periods, which was highly non-significant. Therefore, our study provides no evidence that 30 days of exposure to a warm temperature resulted in greater tolerance to high temperatures than two days of exposure to the same warm temperature.

## Discussion

We evaluated the effects of short- and long-term acclimation to warm temperatures on CT_max_ for *P. cinereus*, with the expectation that extended exposure to warm temperatures would lead to a significant increase in CT_max_. Although salamanders exposed to the long-term acclimation treatment had a CT_max_∼1 °C higher than control salamanders, this difference was not statistically significant, and the difference in CT_max_ between animals experiencing warm temperatures for 30 days *versus* 48 h was slight and highly non-significant. Overall, our results indicate that individual *P. cinereus* exposed to warm temperatures for relatively long periods of time do not experience greater tolerance to high temperatures than individuals experiencing short-term exposure to warm conditions.

Previous studies have evaluated the physiological effects of duration of exposure to warm temperatures in various ectothermic organisms (MacLean et al., 2017a; MacLean et al., 2017b). For example, Cicchino et al. (2023) examined the acclimation effects in tadpole populations from two ascaphid frog species exposed to different holding temperatures, evaluating their acute responses. Their findings revealed notable intra- and interspecific variations in CT_max_: some populations exhibited significant increases in warmer conditions, while others showed greater variability and decreased CT_max_ in acute trials. These findings suggest that exposure time, acclimation temperature, and interspecific differences can strongly influence the physiological responses of ectotherms. In our study, salamanders exposed to long-term treatments may have had more time to fully acclimate, though the differences in CT_max_ between treatment groups and the control were minimal. The slight differences we observed may reflect the varying effects of thermal shock, with longer exposure times potentially extending thermal tolerance compared to the control group (Deery et al., 2021).

Despite the lack of statistical significance among treatments and the control group, there was a small but consistent increase in CT_max_ for the salamanders after exposure to warmer temperatures. Salamanders exposed to short-term treatment may have experienced heat stress in response to the temperature increase, leading to heat hardening due to thermal shock (Yu et al., 1998; Deery et al., 2021; Dallas & Warne, 2023). This response is often mediated by the regulation of heat shock proteins (HSPs) in response to acute thermal stress, as previously evaluated in *P. cinereus* (Yu et al., 1998) and *Anolis* lizards (Deery et al., 2021). Interestingly, it has been reported that the amount of HSP70 in *Plethodon cinereus* does not change significantly based on varying acclimation and hardening treatments. For example, salamanders exposed to cold (2–3 °C), control (15 °C), and heat shock at 2 °C below CT_max_ (∼34.3 °C) did not show a significant change in HSP levels (Yu et al., 1998). This might help explain the minimal change in CT_max_ among the treatment groups and the control in our study. However, in a study of *Anolis* lizards, researchers reported a heat-hardening response for the temperate species *Anolis carolinensis*, but not for the tropical lizard *A. sagrei*, indicating different plastic responses to heat treatments between species (Deery et al., 2021).

Several other factors may also explain the low variation in CT_max_ despite the thermal treatments described in our study. One possibility is that CT_max_ is more phylogenetically conserved across species (Sunday, Bates & Dulvy, 2011), limiting the potential for rapid acclimation. Another possibility is that the temperature changes in our treatments were insufficient to elicit a stronger physiological response. For example, previous studies have applied higher acclimation temperatures on two *Ambystoma* salamanders (∼27 °C, ∼32 °C), resulting in larger increases in CT_max_ (Messerman, Turrell & Leal, 2022). Seasonal acclimation may also play a more critical role in *P. cinereus*, given its native range. For example, Jones et al. (2023) found a significant increase in CT_max_ across active seasons with an increase of ∼5 °C from late Spring-early fall compared to early spring-late fall. Feder & Pough (1975) noted that this species selects different temperatures seasonally, suggesting that longer-term environmental changes may be more influential than rapid temperature shifts. Additionally, the short-term treatment in our experiment might not have allowed enough time for full acclimation, potentially underestimating the acclimation capacity of the Eastern Red-backed Salamander (Rohr et al., 2018).

In contrast to our findings, other studies have demonstrated more pronounced effects of both exposure times and thermal variation on CT_max_ in ectotherms (Davenport & Castle, 1895; Doudoroff, 1942; McFarland, 1955; Stillman, 2003; Young & Gifford, 2013; Markle & Kozak, 2018; MacLean et al., 2017a; Messerman, Turrell & Leal, 2022; Zhao et al., 2022). For instance, one compelling study on the temperate salamander *Desmognathus brimleyorum* revealed a significant increase in CT_max_ under variable and warmer conditions, despite the change being less than 1 °C (Young & Gifford, 2013). This suggests that thermal acclimation capacity may vary widely across ectotherms. Similarly, Markle & Kozak (2018) found a positive correlation between acclimation capacity and CT_max_ increases in *P. cinereus*, reinforcing the idea that thermal plasticity exists in this species but may be more limited in our study due to the specific conditions applied.

Size has also been reported as a factor influencing acclimation capacity, with larger organisms typically requiring more time to fully acclimate than smaller ones (Rohr et al., 2018). To control potential size effects, we conducted an ordered ANOVA including body condition as a predictor, but the results were not statistically significant, indicating that body condition did not influence CT_max_ responses in our study. While size did not seem to affect the results overall, it could still influence acclimation time, as smaller organisms typically acclimate more rapidly than larger ones (Rohr et al., 2018).

Our work contributes to a growing literature that describes how thermal plasticity affects ectotherms in variable environments. Such plasticity may be crucial for survival in the face of climate change, as temperature fluctuations are becoming more pronounced. However, phenotypic plasticity is not the only way for species to respond to changing environmental conditions- behavioral adaptations may also play an important role (Wong & Candolin, 2015). *Plethodon cinereus* has a number of behavioral adaptations that may limit its exposure to dangerously high temperatures, including its primarily nocturnal activity, its use of cool, moist microhabitats (Farallo & Miles, 2016), and the ability to burrow in the soil when conditions are unfavorable (Heatwole, 1960). Thus, although we found no evidence that CT_max_ differed between short-and long-term exposure to a warm temperature in *P. cinereus*, it remains to be seen whether this makes them vulnerable to abrupt changes in their thermal landscape (Deery et al., 2021).

## Conclusions

We tested the hypothesis that prolonged exposure to a warm temperature by the salamander *Plethodon cinereus* results in higher thermal tolerance than short-term exposure to the same high temperature. We found no difference in CT_max_ between individuals experiencing short- and long-term exposure to a high temperature, implying that the species has limited capacity to rapidly acclimate to sudden temperature changes. Future studies would benefit from larger sample sizes and additional acclimation treatments, as well as consideration of behavioral adaptations to more fully assess how exposure to high temperatures affects *P. cinereus*.

## Supplemental Information

10.7717/peerj.20775/supp-1 Supplemental Information 1 R code of the statistical analysis and figures

10.7717/peerj.20775/supp-2 Supplemental Information 2 Raw data of the CTmax, body conditions and variables evaluated in this study

## Additional Information and Declarations

### Field Study Permissions

The following information was supplied relating to field study approvals (i.e., approving body and any reference numbers): Field collection was approved by the Ohio Division of Wildlife.

### Data Availability

The following information was supplied regarding data availability: The raw data and code are available in the Supplemental Files.
